# Beyond Handcrafted Features: A Deep Learning Framework for Optical Flow and SLAM

**DOI:** 10.3390/jimaging11050155

**Published:** 2025-05-15

**Authors:** Kamran Kazi, Arbab Nighat Kalhoro, Farida Memon, Azam Rafique Memon, Muddesar Iqbal

**Affiliations:** 1 Institute of Information and Communication Technologies, Mehran University of Engineering and Technology, Jamshoro 76062, Pakistan; kamran.kazi@faculty.muet.edu.pk (K.K.); arbab.nighat@faculty.muet.edu.pk (A.N.K.); 2 Department of Electronic Engineering, Mehran University of Engineering and Technology, Jamshoro 76062, Pakistan; farida.memon@faculty.muet.edu.pk; 3 Renewable Energy Laboratory, College of Engineering, Prince Sultan University, Riyadh 11586, Saudi Arabia

**Keywords:** CNN optical flow estimation, feature extraction, map building, offset error, visual SLAM

## Abstract

This paper presents a novel approach for visual Simultaneous Localization and Mapping (SLAM) using Convolution Neural Networks (CNNs) for robust map creation. Traditional SLAM methods rely on handcrafted features, which are susceptible to viewpoint changes, occlusions, and illumination variations. This work proposes a method that leverages the power of CNNs by extracting features from an intermediate layer of a pre-trained model for optical flow estimation. We conduct an extensive search for optimal features by analyzing the offset error across thousands of combinations of layers and filters within the CNN. This analysis reveals a specific layer and filter combination that exhibits minimal offset error while still accounting for viewpoint changes, occlusions, and illumination variations. These features, learned by the CNN, are demonstrably robust to environmental challenges that often hinder traditional handcrafted features in SLAM tasks. The proposed method is evaluated on six publicly available datasets that are widely used for bench-marking map estimation and accuracy. Our method consistently achieved the lowest offset error compared to traditional handcrafted feature-based approaches on all six datasets. This demonstrates the effectiveness of CNN-derived features for building accurate and robust maps in diverse environments.

## 1. Introduction

Simultaneous Localization and Mapping (SLAM) is a critical task in robotics [1,2] and computer vision, enabling autonomous systems to navigate unknown environments by simultaneously estimating the robot’s trajectory and building a map [3]. Traditionally, SLAM relies heavily on handcrafted features—such as Scale-Invariant Feature Transform (SIFT), Speeded Up Robust Features (SURF), and Oriented FAST and Rotated BRIEF (ORB)—to extract visual cues from the environment. While these methods have been effective, they exhibit significant limitations under challenging conditions, such as changes in viewpoint, occlusions, and varying illumination conditions.

To overcome these limitations, deep learning techniques, particularly Convolutional Neural Networks (CNNs), offer a promising alternative by learning robust feature representations directly from data. In this paper, we propose a novel deep learning-based approach to SLAM that harnesses the power of CNNs to improve the robustness and accuracy of map estimation. Specifically, we extract features from intermediate layers of three widely used CNN architectures—ConvNeXtXLarge, EfficientNetV2L, and NASNetLarge—and evaluate their effectiveness in reducing odometry error compared to traditional methods.

Our approach involves estimating the odometry error [4] across various layers and filters within these CNNs to identify the feature combination that minimizes the error. We then compare the results with odometry errors calculated using ORB features, which are commonly used in ORB-SLAM, one of the most well-known and widely used SLAM algorithms. The filter combination with the lowest odometry error is subsequently applied to estimate the map of the environment, following the key steps outlined in the ORB-SLAM framework.

The other contributions of the proposed methods include the following:

1. This research uses CNN features to minimize reprojection error in estimating trajectory maps.
2. This gives hope of obtaining minimal reprojection errors without even optimizing the map; hence, the needfor loop closure detection and map optimization can be eliminated in the future.
3. This paper explores the ConvNeXtXLarge, EfficientNetV2L, and NASNetLarge models, performing a detailed layer-wise and filter-wise analysis to identify features that yield minimal odometry error in SLAM tasks.
4. The CNN-based features show improved robustness to common environmental challenges in SLAM, such as viewpoint changes, occlusions, and illumination variations, which often degrade the performance of traditional handcrafted features.
5. The use of CNN features in place of handcrafted features results in a significant improvement by reducing odometry error, which suggests that CNN features can be used to estimate efficient trajectory maps without the need for map optimization in the future.

The experimental results conducted on six publicly available benchmark datasets demonstrate that our deep learning-based feature extraction method significantly reduces odometry error compared to traditional handcrafted features, particularly ORB. This reduction in error not only improves the accuracy of map estimation [5] but also enhances the system’s robustness in the face of environmental challenges such as occlusions, dynamic objects, and changing lighting conditions. Our proposed method represents a step forward in the integration of deep learning [6] with SLAM, offering a more reliable solution for autonomous navigation in diverse real-world environments.

The remainder of this paper is organized as follows: In Section 2, existing work related to the proposed topic is discussed. Section 3 elaborates on the proposed method. It details the analysis of various layers and their underlying filters and methods, information which is used to find the odometry error. Subsequently, we present a comparative analysis with existing SLAM models to highlight the reduction in odometry error achieved by our method in Section 4. Furthermore, we evaluate the proposed approach on six publicly available datasets, encompassing diverse environments and challenges. The extensive evaluation demonstrates the robustness and effectiveness of the proposed approach in real-world scenarios. Finally, we conclude by summarizing the key findings and discussing potential avenues for future research in Section 5.

## 2. Related Work

Simultaneous Localization and Mapping (SLAM) is a crucial task in robotics, enabling robots to build a map [1,7,8] of their environment while simultaneously estimating their location within that map. Traditionally, SLAM has relied on handcrafted features like SIFT or ORB for feature extraction. However, these features have limitations in handling viewpoint changes, illumination variations, and occlusions, which can significantly impact the accuracy and robustness of the generated map.

Recent research has explored the potential of leveraging Convolution Neural Networks (CNNs) for feature extraction in SLAM. CNNs, trained on massive image datasets, learn high-level features that are often more invariant to these challenges compared to handcrafted features. This literature review examines existing research on trajectory mapping with a focus on approaches that utilize features extracted from intermediate layers of CNNs.

Several studies have demonstrated the potential benefits of CNN features for SLAM. The authors of [9] introduced DXSLAM, which utilizes a combination of local and global CNN features to improve map consistency. Similarly, the authors of [10] presented VSO, which integrates semantic constraints obtained from CNNs for enhanced pose and map optimization. These works highlight the ability of CNN features to capture richer and more robust information compared to traditional handcrafted methods.

Lin et al. [11] developed an approach based on object-level semantic mapping and topology-aware features. Their system uses semantic object detection [12,13] and topological relationships to improve loop closure detection [7,14,15]. The topological features improve robustness by abstracting the environment at a higher level than pixel-based or handcrafted feature methods, making it less sensitive to local environmental changes. Chen et al. [16] proposed SuMa++, a SLAM system that incorporates LiDAR-based semantic features for more accurate map building and localization. They use deep learning-based semantic segmentation to extract meaningful features from the environment, which improves the performance of traditional LiDAR-based SLAM. Sualeh and Kim [17] provided a comprehensive survey of semantic SLAM approaches. Their review covers a range of feature extraction methods, from handcrafted features like ORB and SIFT to more recent deep learning-based features. They highlight how the shift towards semantic features has improved loop closure detection and place recognition, especially in dynamic and cluttered environments. Mahmoud and Atia [18] proposed a method that combines semantic segmentation with layout estimation to enhance visual SLAM. Their use of CNN-based semantic features allows the system to better understand the scene, particularly in environments with occlusions or varying lighting conditions. This improves the performance of the visual SLAM system, particularly in challenging indoor environments.

Despite the promising results, challenges remain in utilizing CNN features for SLAM. One key challenge is the computational cost associated with extracting features from CNNs. Compared to handcrafted methods, this process can be computationally expensive, potentially impacting real-time performance, especially on resource-constrained platforms [19]. Additionally, the “black-box” nature of CNNs presents another challenge. Understanding how specific intermediate layers contribute to the robustness of feature extraction remains an open question. Furthermore, selecting layers that provide robust features that are reliable when facing environmental changes such as different viewpoints, different times of the day, and different lighting and weather conditions is a real challenge.

This research addresses these limitations by proposing a novel approach for trajectory mapping. We utilize features extracted from an intermediate layer of a pre-trained CNN for optical flow estimation. Our approach involves an extensive search to identify the optimal layer and filter combination that minimizes offset error while maintaining robustness to viewpoint changes, occlusions, and illumination variations. This strategy aims to achieve accurate and robust map creation in a single session, paving the way for improved SLAM performance in diverse and challenging environments.

Many researchers [20] have created their algorithms based on ORB SLAM [21,22] approaches. The authors of [20] discussed an approach to reducing map points by discarding redundant map points to optimize SLAM. They modified the existing ORB SLAM 2 method and optimized the method for resource-limited hardware platforms.

Various authors [23,24,25] have used landmarks instead of features. According [23], the landmark-based approaches are better for dynamic environments. The authors of [25] made use of detected objects for camera pose recovery in SLAM, with augmented reality being identified as an important application. Their main contributions included the improvement of the re-localization ability of the SLAM system using object landmarks at a higher level. Their system is also able to track and reconstruct objects in 3D by making use of ellipsoids. Experimentation was performed for objects as well as point-based strategies.

A visual SLAM algorithm is presented in [26] that utilizes multi-map fusion to enable fast relocation or local map construction in a scenario where current position and tracking are lost. Fusion of all previous maps is performed during loop back. Moreover, for location recognition, a modified version of the bag of visual words method is used to ensure loop errors are avoided as much as possible, followed by robust map fusion. They did not use bag-of-words-based methods, which are not efficient in complex environments. These cannot match scenes from different viewpoints and in different illumination conditions.

The authors of [27] proposed a robust long-term robotic mapping system using ground segmentation, outlier-robust registration, hierarchical multi-session SLAM, and instance-aware static map building to handle dynamic environments.

## 3. Methodology

The major task of the proposed research in this paper is to find features that are robust, consistent, and reliable in all or in most of natural conditions. However, considering all natural conditions is nearly ideal and not possible in realistic scenario. Almost all other researchers target only a single environmental issue, keeping other natural conditions constant, which makes their algorithms suitable for specific natural conditions, leading to failures in their methods when environmental conditions change.

The proposed method in this paper handles multiple natural conditions like illumination changes, appearance changes, viewpoint changes, and the presence of dynamic objects. The proposed method has an estimated offset error from thousands of filters from various layers of a CNN network and incorporates a layer that can extract features that are much less disturbed by environmental changes and appearance changes. This layer is then used to create a map of the environment.

### 3.1. Selection of Pre-Trained CNN Models for Feature Extraction

Our proposed approach utilizes features extracted from an intermediate layer of a pre-trained Convolution Neural Network (CNN) for optical flow estimation. This is in contrast to traditional SLAM methods that rely on handcrafted features like SIFT or ORB. While handcrafted features have been widely used, they exhibit limitations in handling variations in the environment. For instance, viewpoint changes can significantly alter handcrafted features, leading to inaccurate map creation [28]. Illumination variations and occlusions further hinder these methods [29].

In contrast, CNNs, trained on massive image datasets, learn high-level features that are demonstrably more robust to these challenges [9]. These learned features encode richer information about the scene, leading to improved map consistency [9] and more accurate pose and map optimization [10]. By leveraging CNN features, we aim to achieve robust and accurate trajectory mapping even in environments with viewpoint changes, illumination variations, and occlusions.

To exploit the potential of CNN features, we have to use the features extracted from the CNN to find the optical flow and create a map of the environment. Now, the real challenge is which filter from which layer is most useful and can be reliable in case of scene appearance changes or in case of continuously changing natural conditions. So, to find the answer to this question, we extracted features from multiple filters (i.e., kernels) from various layers of three popular CNN models that are pre-trained on the Imagenet dataset and that can be customized using keras/tensorflow. Out of 38 available CNN models, we have extracted features from three models with unique architectures that have superior Top-1 accuracy. The models that we have picked and their parameters are given in Table 1. The filters that were not used include layers such as the Max Pooling layer, Normalization Layer, Flatten Layer, and other non-convolution layers.

### 3.2. Analysing Filters and Estimating Odometry Error

**Figure 1:**
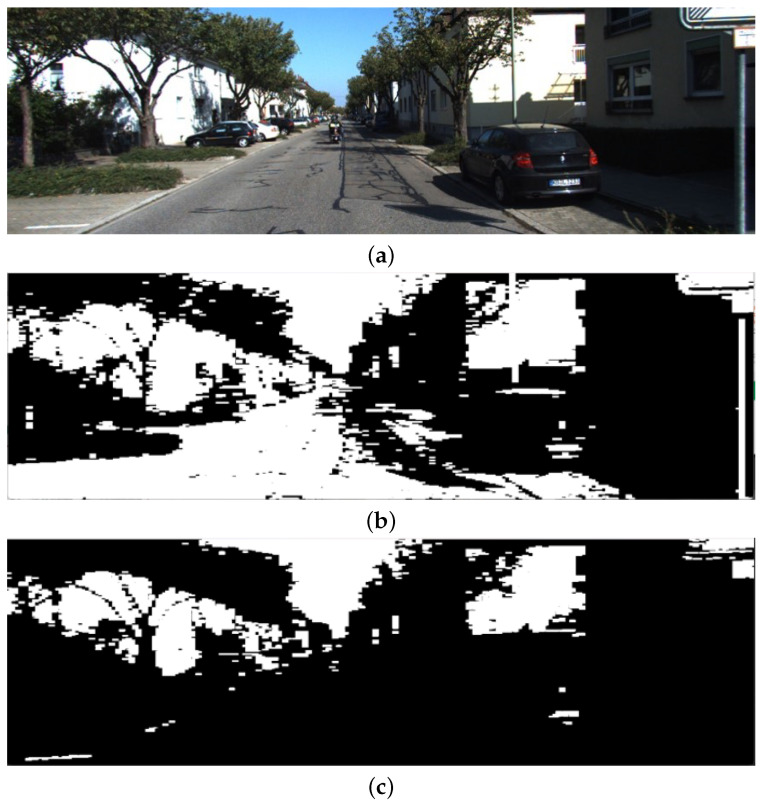

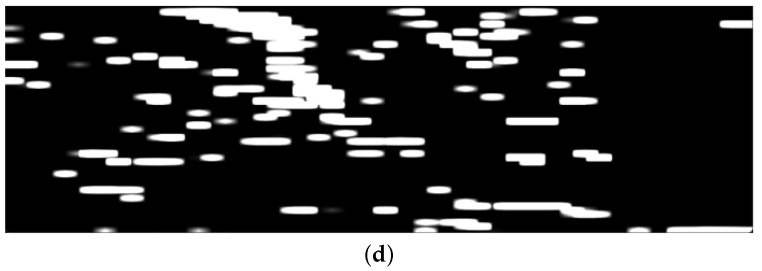
Features obtained from intermediate layer of ConvNeXtXLarge dataset. (**a**) Input image from Kitti Sequence 00; (**b**) image taken from Layer 0, Filter 0 of ConvNeXtXLarge model; (**c**) image taken from Layer 1, Filter 0 of ConvNeXtXLarge model; (**d**) image taken from Layer 3, Filter 0 of ConvNeXtXLarge model.

The convolution layers of each model are analyzed and features from each filter of each convolution layer are extracted to find the odometry error, and the filter with the minimum odometry error is selected for creating the estimated map of the environment. Figure 1 shows the input image in (a), whereas (b), (c), and (d) represent images taken from intermediate layers, resized to the same dimensions as the input to visualise the impact through different layers.

A total of 313,746 filters were analyzed from the three chosen models and compared with the most popular ORB features that are used by many SLAM algorithms for trajectory estimation.

(1) $$E_{t}=\left|\right|T_{c}-T_{p}\left|\right|$$

(2) $$E_{r}=cos^{-1}\left(\frac{trac(R_{c}\cdot R_{p})-1}{2}\right)$$

where $T_{c}$ and $T_{p}$ represent the current translation vector and previous translation vector, respectively. Similarly, $R_{c}$ and $R_{p}$ represent the current and previous rotation matrices. Symbol || represents the Euclidean distance between two vector points and trac represents the sum of diagonals of matrices.

We estimated the odometry errors from 313,746 filters of the three models; some of the filters and their errors are mentioned in Table 2.

The most suitable filter was filter #10 from the convnext_xlarge_stemlayer of the ConvNeXtXLarge model.

### 3.3. Datasets Used for Testing the Proposed Method

The proposed method used six publicly available datasets that are mostly used for validating trajectory mappings of environments obtained using the structure of motion and by estimating optical flow. These datasets include the following:

1. Kitti Sequence 00 [30].
2. Kitti Sequence 02 [30].
3. Kitti Sequence 05 [30].
4. Kitti Sequence 06 [30].
5. Kitti Sequence 08 [30].
6. Kitti Sequence 09 [30].

**Figure 2:**
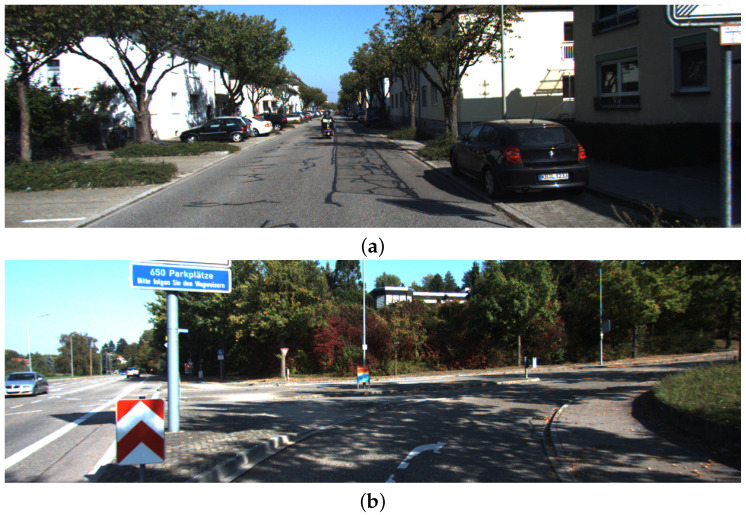

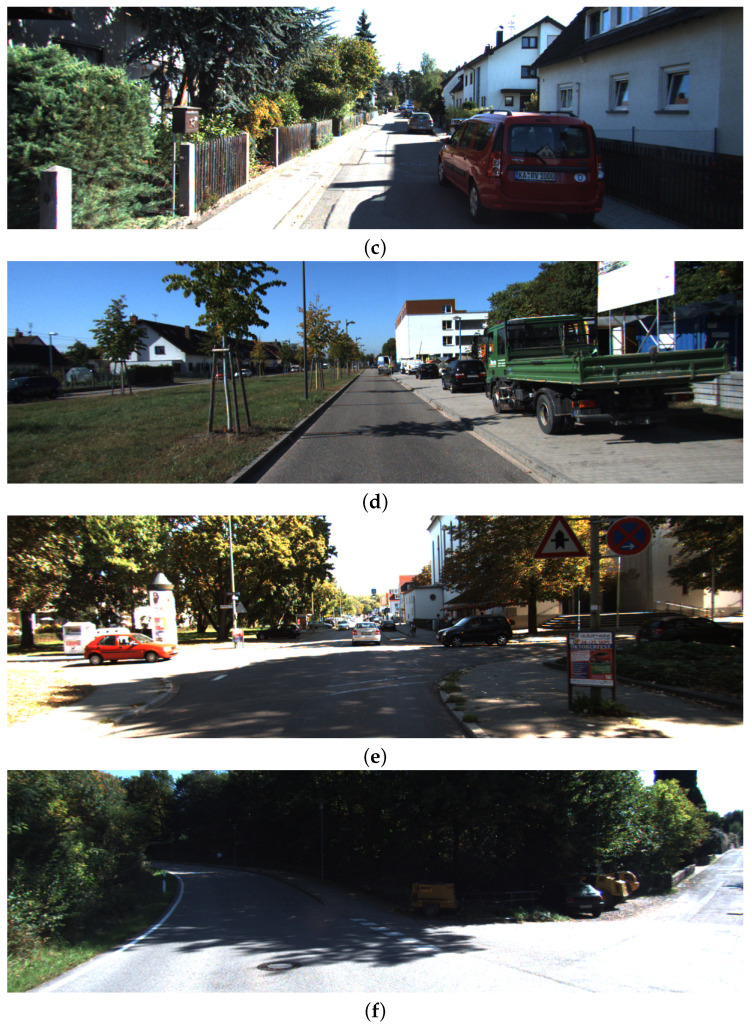
Glimpse of datasets used to validate trajectory mapping. (**a**) Kitti Sequence 00; (**b**) Kitti Sequence 02; (**c**) Kitti Sequence 05; (**d**) Kitti Sequence 06; (**e**) Kitti Sequence 08; (**f**) Kitti Sequence 09.

A glimpse of these datasets is shown in Figure 2 and the details are given below:

#### Kitti Sequence Datasets

The Kitti Sequence Datasets [30] contain 22 stereo sequences, out of which 11 sequences have ground truth available. The sequences were taken in the afternoon time and had various lighting variations and shade levels. Some of the sequences have occlusions, slight viewpoint changes, illumination changes, and the presence of road traffic. The Kitti sequences 00, 02, 05, 06, 08, and 09 are used to validate the proposed approach in this paper, as these sequences have loops, which help in optimizing the maps. More details on the datasets are given in Table 3.

### 3.4. Estimating Transformation Matrix from CNN Features

To create the environment map, it is essential to find the optical flow. The optical flow can determine how a scene is moving. The transformation matrix determines the scene movement. Assume $I_{t}$ is the most recent image frame at current time *t* and $I_{t-1}$ is the previous image frame acquired at previous time t−1. The features acquired from convnext_xlarge_stem layer # 2 and filter # 10 as $F_{I_{t}}$ from image frame $I_{t}$ are compared with features acquired from the same layer and filter $F_{I_{t-1}}$ from previous image frame $I_{t-1}$.

### 3.5. Plotting the Map of the Trajectory

In visual SLAM using CNNs, trajectory mapping is essential for understanding the path estimated through frames captured by the camera, facilitating accurate map creation. This section outlines the process of generating local trajectory maps from sequences of frames, leveraging CNN-derived features to enhance robustness against viewpoint changes, occlusions, and illumination variations. Each sequence contributes to a cohesive trajectory map, refined through feature extraction from intermediate layers of a pre-trained CNN model.

To compute the full trajectory, it is necessary to determine the relative transformation $T_{k}$ between consecutive image frames $I_{t}$ and $I_{t-1}$, where *t* is any time >0. These relative transformations are concatenated to reconstruct the complete path incrementally, pose by pose, as the camera moves through the environment. At regular intervals (every *m* poses), the trajectory is refined to provide a more accurate estimate of the local path. This refinement is achieved by minimizing the offset error using CNN-derived features, enhancing stability in challenging environments. In this approach, the extracted CNN features serve as the basis for robust trajectory mapping, enabling a more consistent and optimized map across a range of viewpoints and lighting conditions.

To plot the trajectory map, optical flow between consecutive frames is calculated using CNN-extracted features. When the initial pose $Pos_{init}$ is unknown, robust CNN-derived features from frames $F_{I_{t}}$ and $F_{I_{t-1}}$ are employed to estimate both the Homography (Equation (3)) $H_{I_{t}I_{t-1}}$ and the Fundamental matrix (Equation (4)) $F_{I_{t}I_{t-1}}$. This process establishes the initial pose, enabling trajectory mapping that remains accurate and consistent as the mapping data accumulate. Through the CNN’s learned features, this approach provides a solid foundation for further enhancements in map optimization and adaptability to dynamic environments, setting a new standard for accuracy and robustness in SLAM applications.

(3) $$I_{t}=H_{I_{t}I_{t-1}}I_{t-1}$$

(4) $$I_{t-1}^{T}F_{I_{t}I_{t-1}}I_{t-1}=0$$

To find out which matrix to use for initial pose estimation [31], we need to find score $S_{M}$, as also carried out by [21].

(5) $$S_{M}=\sum (\rho_{M}\left(d_{I_{t}I_{t-1}}^{2}\left(I_{I_{t}}^{i},I_{I_{t-1}}^{i},M\right)\right)+\rho_{M}\left(d_{I_{t-1}I_{t}}^{2}\left(I_{I_{t}}^{i},I_{I_{t-1}}^{i},M\right)\right)$$

where *M* is either *H* for homography or *F* for fundamental matrix. $d_{I_{t}I_{t-1}}^{2}$ and $d_{I_{t-1}I_{t}}^{2}$ are the symmetric transfer errors [21,32] from one frame to another frame.

(6) $$\rho_{M}\left(d^{2}\right)=\left\{\begin{array}{cc} \Gamma -d^{2} & \mathrm{if}\;d^{2}<T_{M}\\ 0 & \mathrm{if}\;d^{2}\geq T_{M} \end{array}\right.$$

where $T_{M}$ is the outlier rejection threshold based on the $\chi^{2}$ test. Γ is equal to $T_{H}$, so that both models score equally for the same *d* in their inlier region, again to make the process homogeneous [21].

To find which model to use, we use the following [21]:

(7) $$R_{H}=\frac{S_{H}}{S_{H}+S_{F}}$$

When $R_{H}$ > 0.40, the homography matrix is considered to take the lead, which adequately captures the planar and low-parallax cases. Otherwise, the fundamental matrix takes the lead.

#### 3.5.1. Estimating Transformation Matrix from Homography

In the case of the homography matrix, eight motion hypotheses using the method of Faugeras et al. [33] are discussed. Their method proposes various tests to find a valid solution. To find the valid solution, we need to find out whether the feature points found in the image frames at time *t* and t−1 are in front of the camera or if some points go behind the camera in a recent frame at *t*.

(8) $$Z=\frac{(R^{1^{r}}-Fg_{I_{t-1}}\times R^{3^{r}})\cdot T}{\left(R^{1^{r}}-Fg_{I_{t-1}}\times R^{3^{r}}\right)\cdot Fg_{I_{t-1}}}$$

(9) $$P_{1}^{3d}=\left(Fg_{I_{t}}\times Z,Fg_{I_{t-1}}\times Z,Z\right)$$

(10) $$P_{2}^{3d}=\left(R^{T}\cdot P_{1}^{3d}-R^{T}\cdot T\right)$$

The $n^{r}$ above any matrix refers to the *n*-th row of that matrix. $P_{1}^{3d}$ and $P_{2}^{3d}$ represent 3d points; if any of these is negative, the points are not in front of the camera in both frames. *R* and *T* represent the rotation and translation matrix estimated by decomposing the homography matrix.

The initial value is always the current value $R_{c}=R$ and $T_{c}=T$.

(11) $$\left\{\begin{array}{cc} T_{c}=T_{c}+scale*R_{f}\cdot T & \mathrm{if}\;T_{z}>T_{x}\;\&\;T_{z}>T_{y}\\ T_{c}=T_{c} & otherwise \end{array}\right.$$

(12) $$\left\{\begin{array}{cc} R_{c}=R\cdot R_{c} & \mathrm{if}\;T_{z}>T_{x}\;\&\;T_{z}>T_{y}\\ R_{c}=R_{c} & otherwise \end{array}\right.$$

$T_{cx}$, $T_{cy}$, and $T_{cz}$ represent the *x*, *y*, and *z* positions in the estimated map, respectively. For a 2D map, the z-axis can be ignored.

#### 3.5.2. Estimating Transformation Matrix from Fundamental Matrix

When the intrinsic parameters of the camera in both image frames at time *t* and t−1 are known, the essential matrix *E* can be determined from the fundamental matrix *F* using

(13) $$E=K^{T}FK$$

where *K* is the camera calibration matrix with the following intrinsic camera parameters:

(14) $$K=\left[\begin{array}{ccc} fx & 0 & cx\\ 0 & fy & cy\\ 0 & 0 & 0 \end{array}\right]$$

where fx and fy are the focal length of the camera and cx and cy are the center points of the camera.

The essential matrix *E* is then decomposed to obtain singular values *w*, left singular values *u*, and right singular values $v_{t}$.

### 3.6. Generating the Trajectory Map

To create the estimated map, based on Equation (7), either the fundamental matrix *F* or homography matrix *H* will be used. In the case of the homography matrix, the transformation matrices *R* and *T* can be obtained from Equation (12). But in the case where the fundamental matrix is chosen by Equation (7), the essential matrix is obtained and then the essential matrix is decomposed and values *w*, *u*, and $v_{t}$ are obtained. These values will help in finding the transformation matrices *R* and *T* using following equations:

Using the Singular Value Decomposition (SVD) method, the essential matrix *E* is decomposed into singular values and rotation and translation vectors are obtained.

The rotation matrix can be calculated as $R=u\times w\times v_{t}$ and the translation matrix as T=u. To calculate the updated translation and rotation on upcoming frames at t+1, Equations (11) and (12) can be used, respectively.

### 3.7. When Initial Pose Is Already Detected

When the initial pose is already detected, we only need to find the essential matrix from features of the current image frame $I_{t}$ and features of the previous image frame $I_{t-1}$ using $f_{I_{t}}\;E\;f_{I_{t-1}}=0$. The rotation and translation matrices are then determined using the method already discussed in Section 3.6.

## 4. Experimental Results

**Figure 3:**
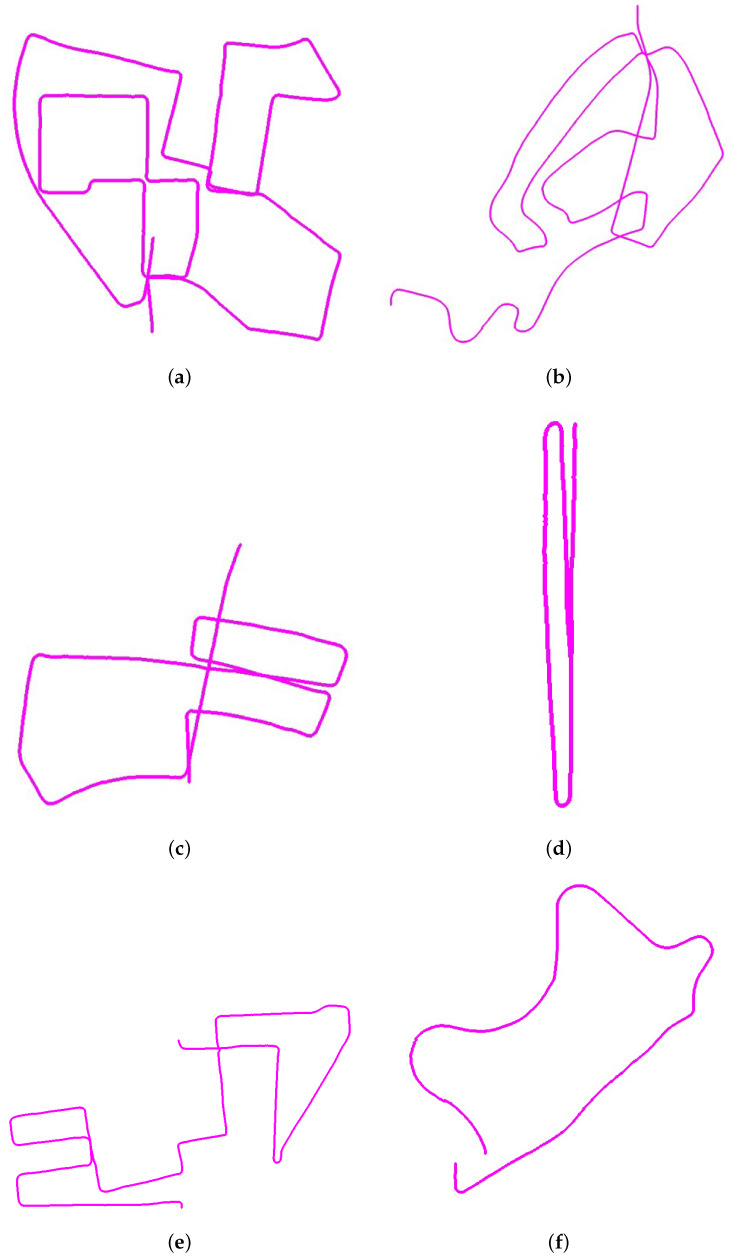
Estimated trajectory maps of Kitti Sequence Datasets without any optimizations. (**a**) Kitti Sequence 00; (**b**) Kitti Sequence 02; (**c**) Kitti Sequence 05; (**d**) Kitti Sequence 06; (**e**) Kitti Sequence 08; (**f**) Kitti Sequence 09.

**Figure 4:**
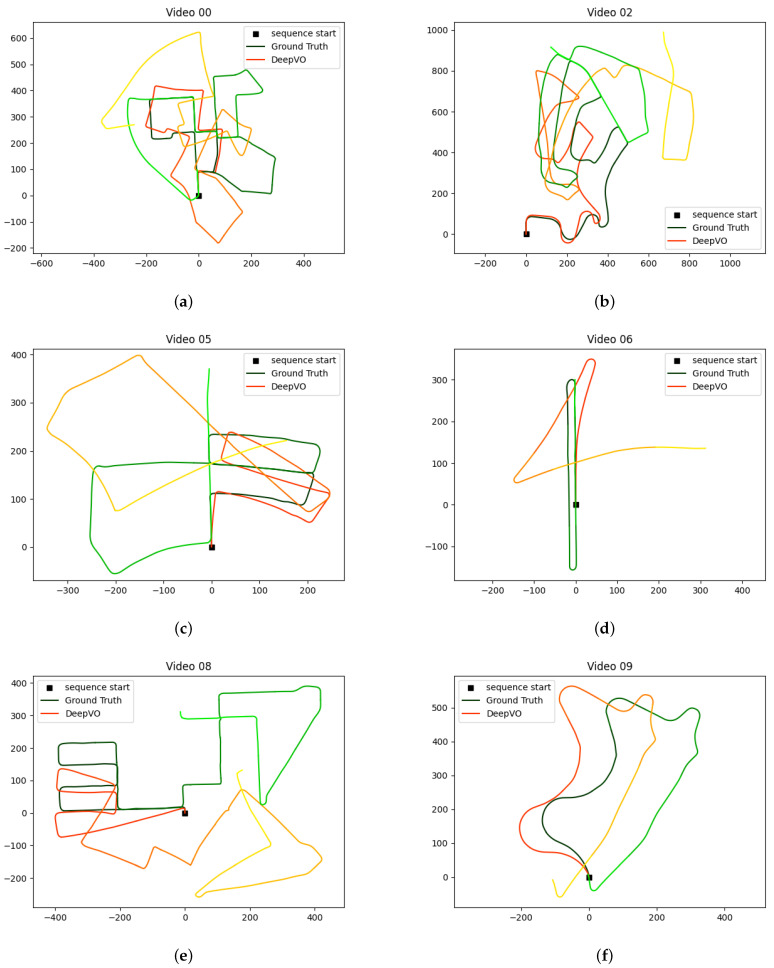
Estimated trajectory maps of Kitti Sequence Datasets using DeepVO [34]. (**a**) Kitti Sequence 00; (**b**) Kitti Sequence 02; (**c**) Kitti Sequence 05; (**d**) Kitti Sequence 06; (**e**) Kitti Sequence 08; (**f**) Kitti Sequence 09.

**Figure 5:**
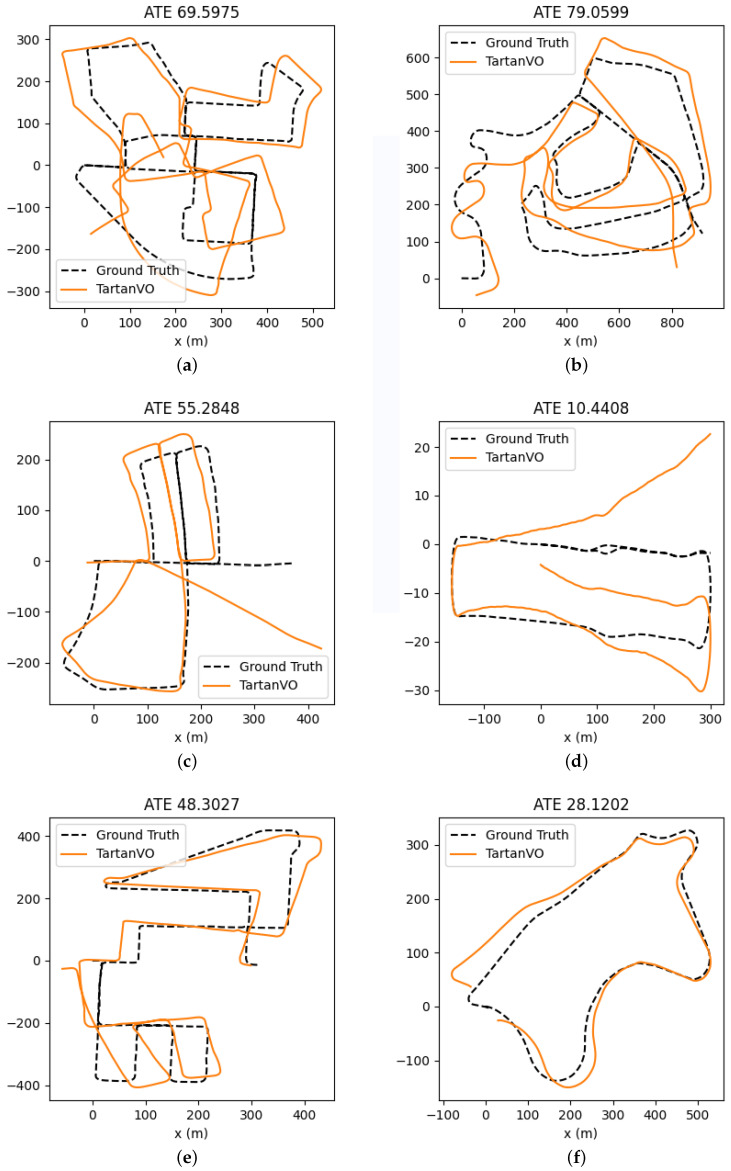
Estimated trajectory maps of Kitti Sequence Datasets using TartanVO [35]. (**a**) Kitti Sequence 00; (**b**) Kitti Sequence 02; (**c**) Kitti Sequence 05; (**d**) Kitti Sequence 06; (**e**) Kitti Sequence 08; (**f**) Kitti Sequence 09.

We now determine the accuracy of the estimated map generated using features obtained from convnext_xlarge_stem layer # 2 and filter # 10. The maps are estimated on six datasets and the estimated trajectories are shown in Figure 3. The proposed method is also compared with two other methods, which also allow for estimating the offset odometry error. The results are listed in Table 4. The estimated trajectory maps of all datasets using Deep-VO [34] are given in Figure 4 and estimated maps using TartanVO [35] are given in Figure 5. The proposed system was tested on an i7-core laptop with 16 GB RAM and it can process from 7 to 13 frames per second.

### Offset Error Estimation on Datasets

To estimate the offset error between the ground truth and the estimated map using proposed method, the following equation is used:

(15) $$E_{x}=\sum ||P_{xg}-P_{xe}\left|\right|$$

(16) $$E_{y}=\sum ||P_{yg}-P_{ye}\left|\right|$$

The offset error in X−axis is $E_{x}$ and the offset error in y−axis is $E_{y}$, which is the sum of all individual frames. Meanwhile, $P_{xg}$ and $P_{yg}$ are the ground truth positions of the current frame in the x−axis and y−axis, respectively, and $P_{xe}$ and $P_{ye}$ are the estimated positions of the current frame in the x−axis and y−axis, respectively.

(17) $$average\;offset\;error=\frac{E_{x}+E_{y}}{N_{f}}$$

where $N_{f}$ is the total number of frames.

## 5. Conclusions

The proposed research introduces a pioneering approach to feature selection in SLAM by conducting an in-depth analysis of layers and filters across three state-of-the-art CNN architectures. This systematic exploration identifies features that exhibit unprecedented robustness to environmental variations—a capability that has not been fully realized in prior SLAM methodologies. Unlike existing approaches, our method achieves significantly lower offset error without requiring map optimization, demonstrating a fundamental shift in how SLAM systems can achieve higher accuracy and reliability. By addressing a long-standing challenge in the field, this work sets a new benchmark for integrating deep learning into SLAM, paving the way for more resilient and precise trajectory mapping in complex environments.

## Figures and Tables

**Table 1:** Models used to find filter with environment-invariant features.

Model	Accuracy		Parameters	Layers	Filters	Used	Not Used
	Top-1	Top-5					
ConvNeXtXLarge	86.7%	-	350.1 Million	297	332,806	36,096	296,710
EfficientNetV2L	85.7%	97.5%	119.0 Million	1031	1,253,254	261,216	992,038
NASNetLarge	82.5%	96.0%	88.9 Million	1041	502,407	16,434	485,973

**Table 2:** Some of the layer and filter combinations and their corresponding estimated translation and rotation errors.

		Average Offset Error	
S. #	Layer Name–Layer #–Filter #	Translation (m)	Rotation (deg)
ConvNeXtXLarge Dataset			
1	Input Layer–00–00	24.54	127.21
2	Input Layer–00–01	32.22	125.14
3	Input Layer–00–02	15.01	108.37
4	convnext_xlarge_stem–2–0	Pose not detected	Pose not detected
5	convnext_xlarge_stem–2–1	442.27	126.01
6	convnext_xlarge_stem–2–2	Pose not detected	Pose not detected
7	convnext_xlarge_stem–2–3	Pose not detected	Pose not detected
8	convnext_xlarge_stem–2–4	31.60	97.35
9	convnext_xlarge_stem–2–5	51.54	130.41
10	convnext_xlarge_stem–2–6	3,580,159.43	104.76
11	convnext_xlarge_stem–2–7	Pose not detected	Pose not detected
12	convnext_xlarge_stem–2 – 8	2809.92	127.59
13	convnext_xlarge_stem–2–9	Pose not detected	Pose not detected
**14**	**convnext_xlarge_stem–2–10**	**7.28**	**78.02**
15	convnext_xlarge_stem–2–11	545,608.38	104.77
16	convnext_xlarge_stem–2–12	Pose not detected	Pose not detected
17	convnext_xlarge_stem–2–13	660,566.05	105.23
18	convnext_xlarge_stem–2–14	Pose not detected	Pose not detected
19	convnext_xlarge_stem–2–15	955,025.13	104.47
20	convnext_xlarge_stem–2–16	4,441,462.76	104.42
21	convnext_xlarge_stem–2–17	Pose not detected	Pose not detected
22	convnext_xlarge_stem–2–18	270,065,492.5	93.92
23	convnext_xlarge_stem–2–19	305,075.56	106.48
24	convnext_xlarge_stem–2–20	Pose not detected	Pose not detected
EfficientNetV2L Model			
1	Input Layer–00–00	10.38	124.41
2	Input Layer–00–01	13.50	113.19
3	Input Layer–00–02	21.73	147.26
4	stem_conv–02–00	293,395.01	105.52
5	stem_conv–02–01	Pose not detected	Pose not detected
6	stem_conv–02–02	Pose not detected	Pose not detected
NASNetLarge Model			
1	input_1 Layer–00–00	4.85	105.52
2	input_1 Layer–00–01	17.36	96.62
3	input_1 Layer–00–02	2,454,372.65	113.61
4	stem_bn1– 02–00	1,149,842.15	110.19
5	stem_bn1–02–01	8.58	96.36
6	stem_bn1–02–02	9.25	105.60

The layer and filter marked in bold shows minimum translation and rotation error.

**Table 3:** Details of datasets used for creating map of environment.

Dataset	No. of Images	Image Size	Ground Truth Available?	Contains Loops?	Widely Used?
Kitti Sequence 00	4541	1241 × 376	Yes	Yes	Yes
Kitti Sequence 02	4661	1241 × 376	Yes	Yes	Yes
Kitti Sequence 05	2761	1226 × 370	Yes	Yes	Yes
Kitti Sequence 06	1101	1226 × 370	Yes	Yes	Yes
Kitti Sequence 08	4071	1226 × 370	Yes	Yes	Yes
Kitti Sequence 09	1591	1226 × 370	Yes	Yes	Yes

**Table 4:** Estimated offset error on datasets.

Dataset	Number of Frames	Average Offset Error		
		Deep-VO [34]	TartanVO [35]	Ours
Kitti Sequence 00	4541	13,227.92	124,361.71	476.40
Kitti Sequence 02	4661	13,005.68	317,924.60	1451.84
Kitti Sequence 05	2761	7250.48	133,977.30	286.65
Kitti Sequence 06	1101	7774.23	45,526.16	310.44
Kitti Sequence 08	4071	23,942.91	203,107.88	374.23
Kitti Sequence 09	1591	4379.55	110,614.15	536.42

## Data Availability

The data used in this paper are already publicly available.
